# Eosinophil percentage elevation as a prognostic factor for overall survival in patients with metastatic renal cell carcinoma treated with tyrosine kinase inhibitor

**DOI:** 10.18632/oncotarget.12126

**Published:** 2016-09-20

**Authors:** Hong-Kai Wang, Fang-Nin Wan, Wei-Jie Gu, Yao Zhu, Bo Dai, Guo-Hai Shi, Hai-Liang Zhang, Ding-Wei Ye

**Affiliations:** ^1^ Department of Urology, Shanghai Cancer Center, Fudan University, Shanghai, China; ^2^ Department of Oncology, Shanghai Medical College, Fudan University, Shanghai, China

**Keywords:** eosinophil percentage, metastatic renal cell carcinoma, tyrosine kinase inhibitor, prognosis, prognostic models

## Abstract

**Background:**

We tried to investigate the prognostic significance of post-treatment eosinophil percentage(Eo %) in metastatic renal cell carcinoma(mRCC) patients undertaking sorafenib.

**Results:**

The median OS for the entire sorafenib treatment period was 21.9 months (95% CI: 17.2–25.9 months). Of the 282 mRCC patients, 101 patients experienced an elevated post-treatment Eo % within two months. Median OS of post-treatment Eo % elevated group and non-elevated group were 42.9 months and 16.8 months(p=0.000). After adding post-treatment Eo % into a modified MSKCC model or Heng's model, 43 and 41 patients were reclassified into favorable group, 5 and 9 patients were reclassified to intermediate group respectively.

**Methods:**

mRCC patients treated with sorafenib from 2006 to 2015 in were evaluated. Pre- and post-treatment Eo % were assessed. Oncologic outcomes were analyzed by overall survival and tumor response rate. Predictive parameters were assessed in a Cox proportional hazard model.

**Conclusions:**

Our study demonstrates that an early elevation of Eo % after sorafenib treatment is a strong predictor of good prognosis. Eo % can be a good supplementary for prognostic models using pre-treatment parameters.

## INTRODUCTION

Renal cell carcinoma(RCC) acounts for approximately 3-4% of all adult malignancies and is the third most common urogenital malignancy in China. Eventually, nearly half of these patients will develop metastatic disease with an poor outcome. Randomized controlled trials have led to the approval of several molecular-targeted agents for the treatment of metastatic RCC [1, 2, 3]. However, before the development of multiple TKIs, interleukin-2(IL-2) and interferon-alfa(IFN-a) based immunotherapy is the only treatment that has been shown to improve survival in metastatic RCC(mRCC) [4, 5], which emphasizes the importance of host immunity in anti-tumor treatments.

So far, systemic inflammatory response markers such as C-reactive protein(CRP) and neutrophil to lymphocyte ratio(NLR) have shown significant prognostic values for mRCC patients [6, 7]. However, there are concerns that nonspecific systemic inflammatory markers may be influenced by acute inflammation or infection and somehow limit their predictive values, thus new markers needed to be evaluated. Eosinophils are traditionally referred to as effector cells in allergic diseases and parasitic infections, who has diverse functions from neutrophils and lymphocytes. It is also known to have endogenous and therapy-induced host responses to cancer [8]. Previous studies have shown an improved prognosis with tumor-associated tissue eosinophilia(TATE) in various types of solid tumors. Eosinophils also have a role in the regulation of the immune response, through antigen presentation to T cells and the production and release of immunomodulatory molecules [9]. Above all, it is interesting whether eosinophil's pre-treatment and post-treatment have predictive abilities in mRCC patients treated with targeted therapy, or whether it can reveal the association between host immunity and clinical outcomes.

In the current study, we tried to investigate the prognostic significance of pre- and post-treatment blood eosinophil cell percentage in mRCC patients undertaking sorafenib.

## RESULTS

### Patient characteristics

The total database included 282 patients with metastatic RCC, of which 200 were male, 82 were female. The demographics and pathological features of the patients were shown in Table 1. The median age was 58 years, ranging from 19-83 years old. Clear cell RCC was present in 233(82.6%) patients, papillary RCC and sarcomatoid RCC were present in 28(9.9%) and 21(7.5%) patients. 219(77.6%) patients underwent a prior nephrectomy, of which only 3 patients had the surgery after targeted therapy. 10 underwent a prior metastasectomy and 51 patients took a prior immunotherapy.

**Table 1:** Patient characteristics (N=282)

Characteristics	No.	%
Sex		
Male	200	70.9
Female	82	29.1
Age, years		
Median	57.3	
Range	19-83	
MSKCC Score		
Low	66	23.4
Intermediate	178	63.1
High	38	13.4
Heng Score		
Low	55	19.5
Intermediate	158	56.0
High	69	24.4
Pathology		
clear cell	233	82.6
papillary	28	9.9
sarcomatoid	21	7.5
Metastatic Sites		
1	131	46.5
2	106	37.6
3	34	12.1
≥4	8	2.8
Prior nephrectomy	219	77.6
Prior metastasectomy	10	3.5
Prior immunotherapy	51	18.1
Best response		
CR/PR	53	18.8
SD	191	67.7
PD	38	13.5
eosinophil >5% before treatment	12	4.1
eosinophil >5% in 1 month of treatment	65	23.0
eosinophil >5% in 2 months of treatment	101	35.0

### Oncologic outcomes

**Figure 1:**
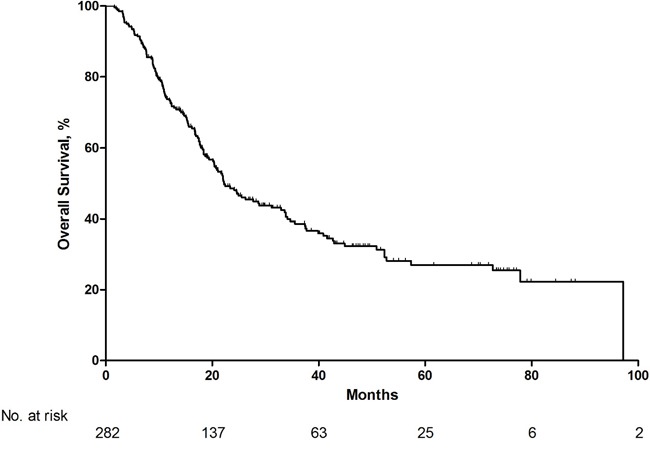
Overall survival for total patients with metastatic renal cell carcinoma

The median OS for the entire sorafenib treatment period was 21.9 months (95% CI: 17.2–25.9 months)(Figure 1). 86 patients undergone a sorafenib dose escalation, 15 exchanged to everolimus, 9 exchanged to sunitinib and 2 exchanged to bevacizumab. CR or PR was achieved in 53(18.8%) patients, SD was achieved in 191(67.7%) patients, PD was achieved in 38(13.5%) patients. At the end of the follow-up, 119 patients were alive with a median follow-up period of 37 months.

**Figure 2:**
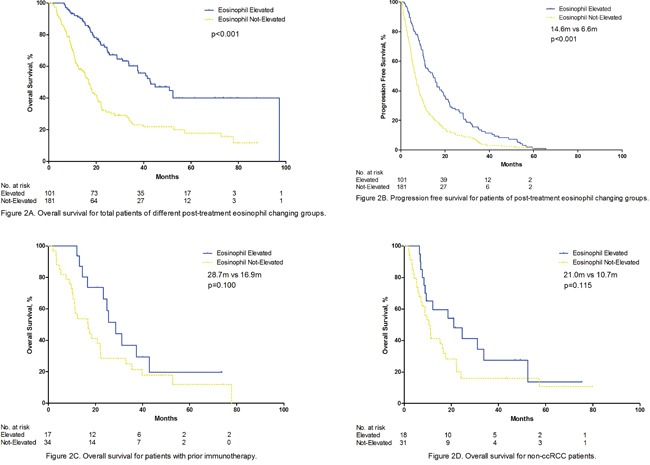
**A.** Overall survival for total patients of different post-treatment eosinophil changing groups. **B.** Progression free survival for patients of post-treatment eosinophil changing groups. **C.** Overall survival for patients with prior immunotherapy. **D.** Overall survival for non-ccRCC patients.

The mean pre- and post-treatment Eo % were 2.06% and 4.74%. 12(4.1%) patients had an elevated Eo %(>5%) before sorafenib, while after treatment 65(23%) experienced an elevated Eo %(>5%) within one months and 101(35.0%) patients experienced an elevated Eo %(>5%) within two months. Median overall survival were significantly different between post-treatment Eo % elevated group(within 2 months) and non-elevated group, the median OS were 42.9 months and 16.8 months(p=0.000)(Figure 2A). Median progression free survival were 14.6 months and 6.6 monhts in both groups (Figure 2B). We did a subgroup analysis of prior immunotherapy treated patients and non-clear cell RCC patients. Although the p value failed to reach 0.05 in these two groups, a trend is observed(Figure 2C and 2D).

**Figure 3:**
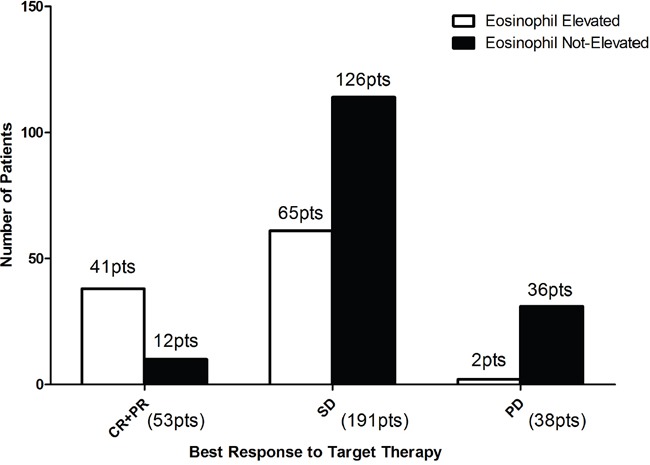
Best response to target therapy for patients of different eosinophil changing groups

**Figure 4:**
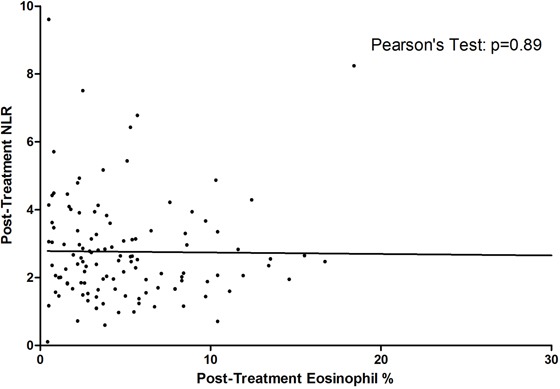
Pearson's test for post-treatment NLR and Post-treatment eosinophil percentage

Univariate analysis showed that KPS≤70, absence of prior nephrectomy, disease free interval≤12 months, metastatic sites≥two, anemia, elevation of serum calcium, elevation of serum lactate dehydrogenase and post-treatment Eo % <5% are associated with poor prognosis. In multivariate analysis, post-treatment Eo % <5% continues to be an independent factor (Table 2). Pre-treatment Eo % is not associated with prognosis. 75.9%, 33.0% and 5.2% patients with CR/PR, SD or PD had an elevated post-treatment Eo %. While 24.1%, 67% and 92.1% patients with CR/PR, SD or PD experienced no elevation of eosinophils. Only 2% patients with elevated Eo % had PD, however nearly 20% patients had early PD whose Eo % didn't elevate(Figure 3). We failed to investigate any association between post-treatment Eo % and other blood cell parameters such as neutrophil count, lymphocyte or NLR(Table 3) (Figure 4).

**Table 2:** Univariate and multivariate analysis for overall survival in patients with mRCC

Characteristics	Category	Univariate analysis			Multivariate analysis		
		OR	(95% C.I.)	p value	OR	(95% C.I.)	p value
Gender	Male vs Female	0.72	0.82-1.65	0.398	-	-	-
Age, years	<64 vs >64	0.82	0.57-1.19	0.289	-	-	-
KPS	>70 vs ≤70	4.39	2.93-6.58	0.000	2.71	1.68-4.19	0.000
Prior Nephrectomy	No vs Yes	0.68	0.46-0.99	0.045	1.54	1.00-2.38	0.017
Disease Free Interval	>12 mo vs ≤12mo	2.76	1.90-3.99	0.000	2.21	1.51-3.39	0.000
Metastatic sites	1 vs ≥2	1.99	1.61-2.47	0.000	1.68	1.34-2.11	0.000
Prior Immunotherapy	No vs Yes	1.41	1.00-2.08	0.052	-	-	-
Anemia	No vs Yes	2.3	1.67-3.18	0.000	1.71	1.20-2.40	0.003
Calcium	<2.5 vs ≥2.5mmol/L	2.08	1.17-3.68	0.012	1.73	0.92-3.24	0.088
Lactate Dehydrogenase	<450 vs ≥450U/L	4.93	2.46-9.81	0.000	3.5	1.91-8.02	0.001
Neutrophil Count	<4.5 vs ≥4.5 ×10^9/L	2.06	1.46-2.92	0.000	1.24	0.86-1.79	0.247
Platelet Count	<300 vs ≥300 ×10^12/L	2.62	1.79-3.83	0.000	1.18	0.75-1.85	0.467
pre-treatment eosinophil %	<5% vs ≥5%	0.6	0.22-1.62	0.315	-	-	-
post-treatment eosinophil %	<5% vs ≥5%	0.37	0.26-0.53	0.000	0.51	0.36-0.75	0.000

**Table 3:** Post-Treatment eosinophil% and its association with other post treatment hematologic parameters

Characteristics	Category	No. of Patients	Post-Treatment Eosinophil% status		
			Not-elevated	Elevated**	p-value
			N=171	N=111	
Neutrophil Count	≥4.5 ×10^9/L	64	46	18	0.062
	<4.5 ×10^9/L	218	125	93	
Lymphocyte	≥0.8 ×10^9/L	263	160	103	0.92
	<0.8 ×10^9/L	19	11	8	
Post-treatment NLR	<3	190	114	87	0.631
	≥3	92	57	34	

** Patients with elevated pre-treatment eosinophils were characterized to elevated group.

### Model reclassification

**Figure 5:**
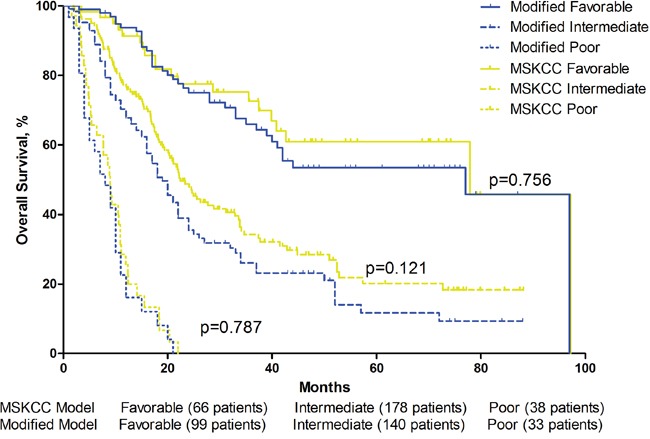
Overall survival by MSKCC Model and modified MSKCC Model risk groups

**Figure 6:**
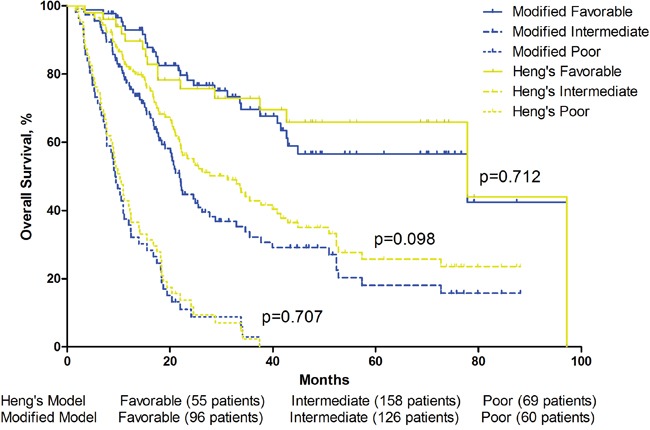
Overall survival by Heng's Model and modified Heng's Model risk groups

After adding post-treatment Eo % into a modified MSKCC model or a modified Heng's model, patients were reclassified into new risk categories. 43 and 41 patients were reclassified into favorable group from intermediate group in the MSKCC and Heng's model respectively, 5 and 9 patients were reclassified to intermediate group from poor group respectively. There were no difference in overall survival of either three risk-groups between the original model and the modified model(Table 4)(Figure 5)(Figure 6). The C-index of the origin MSKCC and Heng's model in our cohort is 0.694 and 0.705, when adding Eo % to the models, the C-index turned to 0.729 and 0.733 respectively.

**Table 4:** Comparison of MSKCC and Heng's Model and their modified models

	MSKCC Model				Post-treatment Eosinophil modified MSKCC Model				
	Patients (No.)	1-Year Survival	2-Year Survival	Median Survival	Patients (No.)	1-Year Survival	2-Year Survival	Median Survival	p-value
Favorable	66	91.40%	77.60%	77.9 months	99	93.70%	76.20%	77.9 months	0.756
Intermediate	178	77.30%	48.80%	22.4 months	140	70.20%	38.60%	19.4 months	0.121
Poor	38	25.10%	0.00%	8.9 months	33	22.60%	0.00%	8.8 months	0.787
	Heng's Model				Post-treatment Eosinophil modified Heng's Model				
	Patients (No.)	1-Year Survival	2-Year Survival	Median Survival	Patients (No.)	1-Year Survival	2-Year Survival	Median Survival	p-value
Favorable	55	86.60%	75.70%	77.9 months	96	92.90%	78.20%	77.9 months	0.712
Intermediate	158	82.20%	55.20%	31.1 months	126	77.50%	44.70%	21.9 months	0.098
Poor	69	41.30%	13.70%	10.3 months	60	35.70%	11.00%	9.4 months	0.707

## DISCUSSION

These results are the first to indicate that an early elevation of Eo % post sorafenib treatment is a strong predictor of good prognosis. Eosinophil elevation can also be a predictor of tumor response to sorafenib. We also observed that post-treatment eosinophil can be a good supplement for the MSKCC and the Heng's score(which is mainly using pre-treatment parameters) to help better stratify patients into different risk groups.

Previous studies had laid much emphasis on cancer progression and host systemic inflammatory response. It is believed that increasing tumor burden or aggressive tumor biology is related to either pro-inflammatory state with over production of cytokines or diversely an immune suppression. Many pretreatment blood-based measurements of systemic inflammatory response are prognostic in patients with RCC, including NLR [6], CRP [7], MLR, GPS [12], granulocyte-to-dendritic cell (DC) ratio [11], neutrophilia [12], thrombocytosis, lymphopenia [13] and so on. CRP is a representative marker of systemic inflammatory response. It is believed that elevated CRP level predicts poorer survival of mRCC patients [14][7]. NLR can reflect the combined prognostic information of both neutrophils and lymphocytes, pre-treatment NLR is an independent prognostic factor for patients with either non-metastatic or mRCC patients [6, 15]. Moreover, a decreased NLR after target therapy is often associated with a better prognosis [16]. Above all it is sure that host systemic inflammatory response may be altered by theraputic intervention. However, factors such as CRP and NLR are systemic inflammatory expressers, we still need another easily achived factor which can reveal host immune enhancement. Afterall we started this retrospective study focusing on Eo %. Considering that a common toxicity of TKIs such as sunitinib is a decrease of blood cell counts, we chose sorafenib to minimize hematological toxicities associated bias.

Eosinophilic granulocytes (eosinophils) constitute less than 5% of the total white blood cells [17]. In the current study we defined a eosinophil cell count more than 5% as an elevation of Eo % and found some association between post-treatment Eo % (one-two months after target therapy) and mRCC outcome. The prognosis of patients who experienced an Eo %elevation early after sorafenib treatment is better than those who did not. More CR and PR were achieved in the eosinophil elevated group indicating that it is a good interpreter of tumor response. To investigate whether the eosinophil change is subsequent to the changes in other hematologic parameters, we compaired post-treatment eosinophil % status to post-treatment neutrophil count, lymphocyte and NLR, and found none statistical association.

To date, MSKCC model and Heng model are the two generally accepted prognostic scoring systems for survival of metastatic RCC patients [18, 19]. However, clinical parameters included in the two models were all pre-treatment parameters, they lack the capability to find patients who were congenital resistant to target agents neither can they provide enough prognostic information during the therapy. Thus lots of patients in the same stage turned out to be quite different in prognosis. During multivariate analysis of our data, we confirmed that post-treatment elevation of eosinophil, KPS, disease free interval, metastatic sites [20], low hemoglobin, serum calcium and lactate dehydrogenase were associated with adverse outcome independently. We didn't find any prognostic value of pre-treatment eosinophil. After we added post-treatment eosinophil to the MSKCC model and the Heng's model, we noticed that many patients were reclassified to lower risk groups with a minor change in median overall survival of each risk group. We then calculated the C-index of the original models and the modified ones and finally observed that post-treatment eosinophil can enhance the predictive accuracy of the predictive models. These results finally indicated that post-treatment parameters can act as a good supplementary in predictive models.

It remains unknown how the elevation of Eo % is correlated to tumor response and survival in mRCC patients taking sorafenib. So far, results about eosinophil and RCC outcome were mainly from interferon-alpha 2b (IFN) or interleukin-2 studies. Wersall first observed an eosinophil elevation in immunotherapy non-responders [21]. Moroni again said that blood eosinophilia predicts a poor response to immunotherapy in patients with advanced RCC [22]. However Lissoni indicated that lymphocyte and eosinophil mean number was significantly higher in patients with PR or SD than in the progressed ones [23], and in another study Rodgers et al found a significant correlation (P < or = 0.05) between eosinophil count and a better survival but not with clinical response [24]. None of the studies used Eo % as a clinical parameter, so the results remain controversal. Yet in a mouse graft study, there is a substantial decrease in both tumorigenicity and tumor progression concomitant with an abundant tumor eosinophilia [25]. The elevation of Eo % observed in our study may be due to tumor necrosis induced eosinophil migration, because it is believed that TATE may develop early and persist throughout tumor development [26, 27]. Indeed, apart from decreased CRP and NLR after treatment, we observed an elevated eosinphil percentage after treatment which indicated different mechanisms in predicting outcome.

There is still some limitations of our study. The sample size in our study is relatively small, we need larger multicenter results to validate our data. We retrospectively used patients undergoing sorafenib therapy, further studies for patients receiving sunitinib or other first line target therapies are needed. More molecular mechanisms about eosinophil and RCC outcome needed to be elucidated.

## MATERIALS AND METHODS

### Patients and parameters

Metastatic RCC patients treated with sorafenib during 2006 to 2015 in our institute were enrolled in the study. At least one measurable tumor lesion was required for each patient according to the Response Evaluation Criteria in Solid Tumors (RECIST, version 1.1) [28]. Pathology was confirmed by prior nephrectomy or tumor biopsy. Histological subtypes included clear-cell (cc) RCC, papillary RCC and ccRCC with sarcomatoid component. All patients were required to undergo blood tests at baseline and every two weeks after initiation of therapy to evaluate renal and hepatic function and check for blood cell counts. CT scans were repeated every eight weeks after initiation of sorafenib to evaluate tumor response. The study protocol was approved by our hospital's Institutional Review Board. All patients provided written informed consent to participate in the study.

The following were included for analysis as binary variables: Gender, age(cutoff=64 years old), karnofsky performance score(KPS)(cutoff=70), prior nephrectomy, disease free interval(cutoff=12months), metastatic sites, prior immunotherapy, hemoglobin level(cutoff=130g/L for male, 115g/L for female), calcium(cutoff=2.5mmol/L), lactate dehydrogenase(cutoff=450U/L), neutrophil count(cutoff=4.5×10^9/L), platelet count(cutoff=300 ×10^12/L), pre-treatment Eo %(cutoff=5%), post-treatment Eo %(cutoff=5%), MSKCC score [18], HENG score [19], post-treatment NLR(cutoff=3). The 5% cut-off value of Eo % is determined according to the reference interval given by Chinese Department of Health. Post-treatment Eo % escalation was counted by the Eo % four-eight weeks after initiation of sorafenib. An Eo % > 5% is defined as "Escalation of Eo %".

The duration of OS was determined from the date of first dosage of sorafenib treatment to death or the date of the last follow-up visit for patients still alive. Disease control was defined as stable disease(SD), a partial response (PR) or a complete response(CR) according to RECIST criteria, the best tumor response will be recorded.

### Model reclassification

As proposed by the NCCN guideline, the MSKCC Model and the Heng's Model are reliable prognostic models for survival prognosis. Hence we used those two models to evaluate the value of post-treatment Eo % in clinical practice. Because an elevated post-treatment eosinophil showed significant prognistic value during the study, and a post-treatment parameter is different from those existing pre-treatment ones(such as KPS, calcium, hemoglobin et al.), we assume the elevation of post-treatment eosinophil can neutralize one of the adverse factors from those prognostic models. For example, if a patient have three adverse factors from Heng's model and then he experienced elevated eosinophil after treatment, he will be counted as having 3-1=2 factors in a modified Heng's model. On the other hand, if a patients have 0 adverse factors at the first time, he will still be counted as having zero factors in a modified Heng's model no matter having an elevated eosinophil or not.

### Statistical analyses

Continuous data are presented as median (range) and binary data are presented as proportions. Association between variables was evaluated on the basis of the chi-square test. Pearson's test was used to determine the association between post-treatment NLR and Post-treatment Eo %. The Kaplan-Meier method was used to determine overall survival rate. Overall survival rates were compared using the log-rank test. Predictive parameters were assessed in a Cox proportional hazard model, and odds ratios with 95% confidence intervals were calculated. The predictive accuracy of the model was evaluated by the concordance index(C-index), which is the area under the receiver operating characteristic curve for censored data, in which a value of 0.5 indicates no predictive discrimination, and a value of 1 represents a perfect ability to separate patients [29]. The C-index analysis was calculated by using the R software. All other analyses were conducted using SPSS 20.0 software (IBM, Chicago, IL, USA). P values were two-tailed and a P < 0.05 was considered to indicate statistical significance.
